# Effects of Peanut Processing on Masticatory Performance during Variable Appetitive States

**DOI:** 10.1155/2010/487301

**Published:** 2009-11-19

**Authors:** Fiona McKiernan, Richard D. Mattes

**Affiliations:** Department of Foods and Nutrition, Purdue University, West Lafayette, IN 47907-2059, USA

## Abstract

*Background*. Current evidence indicates that peanut consumption reduces cardiovascular disease risk, while posing little threat to positive energy balance. However, questions have been raised as to whether inter- and intraindividual variability in mastication in response to peanut form and processing properties may influence these health effects, since mastication has the potential to alter the bioaccessibility of nutrients within the nut matrix. *Objective*. To explore the relationship between peanut form and processing and masticatory function. *Subjects/Methods*. Thirty nine adults (16 M, 23 F; BMI: 30.4 ± 4.0 kg/m^2^; age: 27 ± 8 y) with healthy dentition chewed four different forms of peanuts until they would normally swallow and then expectorated the bolus. Surface electromyograms (EMGs) were obtained from the masseter and temporalis muscles during chewing of the four test foods. The maximum and mean bite forces, duration of chewing sequence, number of chews, and total muscle work for the complete chewing cycle were measured on the integrated EMG in fasted and sated states. *Results*. While no significant differences were noted in response to appetitive state, peanut form and processing had a significant influence on masticatory efficiency, as measured by proportional particle size distributions. The processed peanuts (honey roasted, roasted salted, and roasted unsalted) were chewed significantly fewer times compared to the unprocessed form (raw). Further, the proportional particle sizes within the swallowing bolus were significantly larger for the processed forms compared to the unprocessed form. *Conclusion*. These observations may have implications for bioaccessibility of energy and cardioprotective nutrients as well as endocrine responses, following peanut consumption.

## 1. Introduction

Epidemiological and clinical data indicate that peanut and tree nut consumption reduces cardiovascular disease risk (CVD), while having limited effects on energy balance [1–5]. The high unsaturated fatty acid composition and the presence of components such as antioxidants and plant sterols have been proposed as mediating the beneficial, heart-healthy effects [6]. However, the bioaccessibility of these components, as well as macronutrients, is affected by the integrity of the nut cell wall [7, 8]. This structural component is largely resistant to enzymatic and microbial degradation in the gut and, thus, acts as a physical barrier to the release of intracellular components [8, 9]. If the cell wall is not ruptured, cardioprotective and energy-yielding compounds, such as lipids and lipid soluble antioxidants, are lost in the feces [8, 10–12]. Mastication can rupture cell walls and improve the bioaccessibility of cellular contents [7, 8]. However, there are substantive inter- and intraindividual differences in mastication [13–16], raising questions as to whether differences in chewing efficiency may alter the health effects mediated by peanuts and tree nuts by altering the bioaccessibility of the healthful components they contain.

The variability in chewing responses to food stimuli may be related to differences in palatability [17–20]. Generally, an increase in meal palatability is associated with an increased ingestion rate. The appearance, odor, taste, and texture of foods are altered to enhance their appeal. But, in so doing, this may also alter the efficiency of their mastication and the bioaccessibility of their intracellular components, thereby modifying their potential influence on health. To date, examination of this issue has been limited to almonds [16]. However, peanuts, particularly processed forms, are the most widely consumed nut (technically a legume) in the US [21]. Due to this popularity, and the fact that their sensory properties vary markedly from that of almonds, determining whether the findings with almonds generalize to peanuts is warranted. 

The present study assessed the effects of roasting and the addition of salty and sweet tastes to peanuts on masticatory parameters and preswallowing proportional particle size distribution. It was hypothesized that processed peanuts would have higher palatability ratings, and this would increase the chewing rate and reduce chewing time, leading to larger proportional particle sizes in the preswallowed bolus. An additional hypothesis was that changes in peanut form/texture in response to processing would also alter the chewing efficiency compared to the nonprocessed variety. As differential responses in masticatory performance have also been noted with variations in appetitive state [16, 18], albeit inconsistently [22], the effects of changes in appetite were also explored.

## 2. Methods

### 2.1. Participants

Thirty-nine healthy adults (16 males, 23 females; body mass index (BMI): 30.4 ± 4.0 kg/m^2^ (range: 25.2–42.4 kg/m^2^); age: 27 ± 8 y (range: 19–48 y)) were recruited through public advertising. Eligibility criteria included natural dentition, no acute or chronic diseases, and no legume/nut allergies. Participants were all nonsmokers and were not taking medications reported to affect the study outcome measures. Each participant signed an informed consent form approved by the Purdue University Institutional Review Board.

### 2.2. Experimental Design

A within-subject experimental design was used. After a 10-hours overnight fast, and 1-hour after their habitual breakfast time, participants reported to the laboratory. Height and body weight were measured and appetitive sensations were rated using 100 mm visual analogue scales (VASs) (developed by W. Horn), recorded on a hand-held Personal Digital Assistant (PDA, PalmZire 21, Palm, Inc., Sunnyvale, CA). This electronic appetite rating system posed twelve standardized questions about appetite, such as “How strong is your feeling of hunger right now?” Each VAS was end-anchored with opposing statements of “not at all” to “extremely”. Chewing efficiency and proportional particle size distribution assessments were determined for a series of four test peanuts (whole raw unsalted (skin removed), whole roasted unsalted, whole roasted salted, and whole honey roasted (Golden Peanut Company, GA, USA)) in this state of high hunger. The testing of each peanut was conducted in a randomized order and all on the same test study day. The palatability of each peanut was rated on a 100 mm VAS, anchored with “extremely unpleasant” at one end and “extremely pleasant” at the other end. Participants were then instructed to consume 200 mL of orange juice and a quantity of instant oatmeal required to reach a state of “comfortably full.” This was designated as a 6- on a 9-point scale relating to fullness, with 1 = “not at all” and 9 = “extremely.” The palatability of the meal was rated and appetitive sensations recorded following consumption. Afterwards, chewing efficiency and proportional particle size distributions were measured for the same four peanut test foods in the sated state. With all testing conditions remaining constant, each participant repeated the protocol, separated by at least seven days, and the mean of the two testing days was used for analyses. Participants were requested to maintain activity levels at a stable intensity between and on the testing days.

### 2.3. Texture Analysis

Texture analysis of each of the four peanut forms was conducted using a TA XT2 Texture Analyzer (Stable Micro Systems, Godalming, UK), fitted with a knife probe, set up to record the force used to penetrate the sample to a depth of 3 mm at a speed of 2 mm/sec. Twenty replications were performed for each test peanut type and a mean value calculated.

### 2.4. Chewing Efficiency

The microstructure of chewing was characterized by recording electromyographic (EMG) signals from the masseter and temporalis muscles (BioPac Systems, Inc., Goleta, CA, USA). Five parameters were quantified: maximum bite force (volts), mean bite force (volts), total number of chews, chewing sequence duration (seconds), and the total muscle work (area of the EMG signal). Chewing rate was calculated as the total number of chews per second. 

The four different peanut samples were presented in duplicate, in a randomized order. Participants were instructed to chew each sample, on their dominant chewing side, until they would normally swallow, during which time the EMG activity was recorded. Each sample was then expectorated. Participants rinsed thoroughly between samples with 20 mL aliquots of deionized water.

### 2.5. Proportional Particle Size Distributions

Proportional particle size distributions of the chewed peanut samples were determined by sieving the expectorated boluses through stacks of preweighed sieves with graded mesh size (>3.35 mm, 3.35–2.00 mm, 2.00–1.00 mm, 1.00–0.50 mm, 0.50–0.25 mm, 0.25–0.125 mm, 0.125–0.63 mm, 0.63–0.32 mm and <0.32 mm (WS Tyler, Mentor, OH, USA)). The expectorated samples were rinsed with deionized water (250 mL) and then dried at 55°C (130F) for 6 hours, a time and temperature combination that has been shown to eliminate all water from similarly sized nuts [23]. Each dried fraction was expressed as a percentage of the original dry weight (percent yield). It was assumed that the difference between the percent recovered in the sieves and the original sample was attributable to particles that were smaller than 0.032 mm, since when all of the liquid was collected and dried in a subgroup of individuals (*n* = 3), recovery was 92%–97% of the initial load. These figures are comparable to the yield reported previously after a similar procedure was conducted with expectorated almond samples [16].

### 2.6. Statistical Analyses

Statistical tests were conducted using the Statistical Package for the Social Sciences (SPSS) (version 16.0, Chicago, IL, USA). The effects of time (fasted versus sated) and time × peanut form on chewing efficiency parameters and percent yield in the sieves (proportional particle size) were assessed using repeated-measures analysis of variance (ANOVA). When significant time × peanut form interactions were observed, paired *t*-tests were used for *post hoc* analyses with Bonferroni adjustments. To assess the proportional particle size distribution within sieves for each nut type, a time × peanut form × sieve interaction was explored. Pearson correlation coefficients were calculated to quantify relationships among masticatory parameters and percent yield, appetitive state, and test food palatability. The reliability of the masticatory outcome variables was assessed by the intraclass correlation coefficient. The criterion level for statistical significance was set at *P* < .05 (two-tailed). All data are expressed as mean ± standard deviation (SD).

## 3. Results

### 3.1. Appetitive Ratings

There were no statistically significant differences in appetitive sensations between test days. Hunger ratings were significantly lower during the sated session compared to the fasted session (*F*(1,38) = 169.9, *P* < .01), while fullness ratings were significantly greater (*F*(1,38) = 205.9, *P* < .01).

### 3.2. Palatability of the Peanuts

Hedonic ratings, in millimeters from the extremely unpleasant anchor point, of the honey roasted, roasted salted, roasted unsalted, and raw peanuts were 75(±22), 73(±15), 60(±18), and 34(±24), respectively. Honey roasted and roasted salted palatability ratings were significantly higher than for roasted unsalted and raw nuts (*P* < .05). Palatability ratings for the peanuts did not differ between chewing sessions.

### 3.3. Texture Analysis of the Peanuts

The breaking force was the highest for the raw peanuts (3046 ± 380 gm), followed by honey roasted (1834 ± 232 gm), roasted unsalted (1545 ± 337 gm), and roasted salted (1195 ± 289 gm).

### 3.4. Chewing Efficiency

There was no significant time or time × peanut form interaction for maximum bite strength. There was no time or time × peanut form interaction for mean bite force. Further, mean bite force was not significantly different between the test peanuts in either appetitive state.

**Figure 1:**
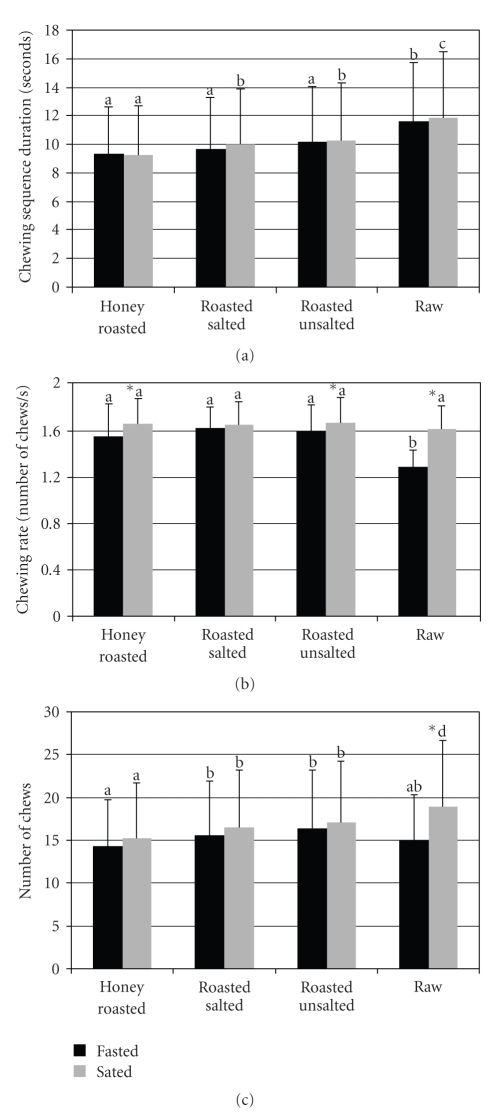
Chewing sequence duration (a), chewing rate per chewing sequence (b), and total number of chews (c) by peanut form and appetitive state test session obtained by electromyographic (EMG) recordings. Values are means ± SD. Letters that are different denote significant differences between peanut forms within the fasted and sated sessions (*P* < .05). *Denotes significant differences between fasted and sated sessions within the peanut form (*P* < .05).

The duration of the chewing sequence did not change significantly between the fasted and sated states and there was no time × peanut form interaction (Figure 1(a)). In the fasted and sated states significantly more time was taken before deglutition for raw nuts compared to the other nut forms (*P* < .01). In the sated state, the duration of the chewing sequence was significantly shorter for honey roasted compared to the other nut forms (*P* < .01).

There was a significant time and time × peanut form interaction for chewing rate (*P* < .01) (Figure 1(b)). The chewing rate was significantly faster in the sated state compared to the fasted state (*P* < .05). The chewing rate increased significantly for honey roasted, roasted unsalted, and raw (*P* < .05) in the sated state, but did not change for roasted salted. The chewing rate was significantly slower for raw peanuts in the fasted state compared to the other nut forms (*P* < .05). However, there were no significant differences between the nut forms in the sated state.

The total number of chews was significantly greater in the sated state compared to the fasted state (*P* < .01) (Figure 1(c)). Further, there was a significant time × peanut form interaction for the number of chews (*P* < .01). The number of chews increased significantly in the sated state compared to the fasted state for raw peanuts (*P* < .01). There was a trend for the number of chews to increase for honey roasted and roasted unsalted nuts in the sated state, but this failed to reach significance (*P* = .07 and *P* = .054, abbreviate resp.). In the fasted state, the total number of chews was significantly lower for honey roasted compared to roasted salted and roasted unsalted (*P* < .01) peanuts but was not significantly different from raw peanuts. In the sated state, significantly fewer chews were observed for honey roasted nuts compared to the other nut forms (*P* < .01) and significantly more chews were made for raw peanuts than the other nut forms (*P* < .01). 

**Figure 2:**
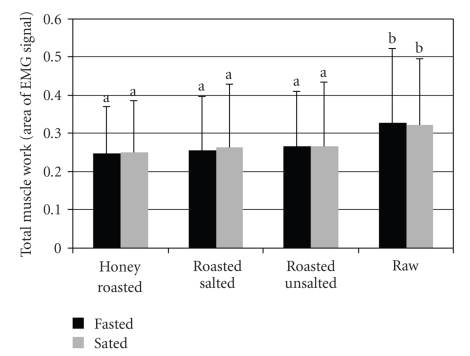
Total muscle work per chewing sequence by peanut form and appetitive state test session obtained by electromyographic (EMG) recordings. Values are means ± SD. Letters that are different denote significant differences between peanut forms within the fasted and sated sessions (*P* < .05).

There was no significant time or time × peanut nut form interaction for the total muscle work (Figure 2). However, under fasted and sated conditions, the total muscle work was significantly greater for raw peanuts compared to the other nut treatments (*P* < .05).

### 3.5. Proportional Particle Size Distributions

**Figure 3:**
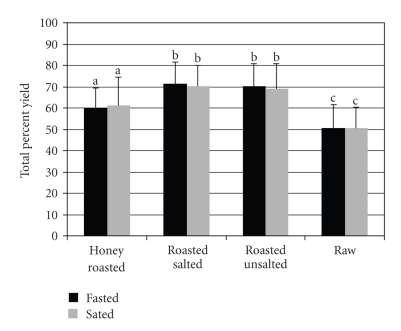
Total percent yield from the expectorated bolus by peanut form and appetitive state test session. Values are means ± SD. Letters that are different denote significant differences between peanut forms within the fasted and sated sessions (*P* < .05).

The overall percent yield (percentage of the original weight recovered) during the fasted (63.0 ± 9.4%) and sated (62.7 ± 9.6%) states was comparable for all peanut forms (Figure 3). Further, there was no significant time × peanut form interaction for the overall percent yield. However, there was a significant peanut form effect (*P* < .01). Regardless of the fasted or sated state, the overall percent yield was significantly lower for raw nuts compared to all other nut treatments (*P* < .01).

**Figure 4:**
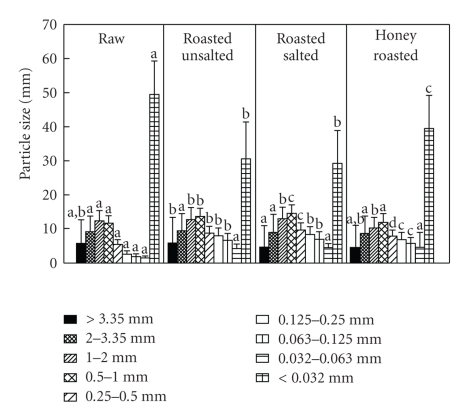
Mean of the fasted and sated particle size distributions by peanut form (percent yield). Values are means ± SD. Letters that are different within the same column denote significant differences between peanut forms (*P* < .05).

There was no significant time or time × peanut form × sieve interaction for particle size distribution (Figure 4). However, particle size distribution did differ significantly according to peanut form (*P* < .01). There were significantly more raw peanuts in the smallest sieve compared to the other three nut forms (*P* < .01).

In the fasted and sated states, the percent yield was significantly and inversely correlated with total muscle work (*r* = −0.38 and −0.39, *P* < .05), number of chews (*r* = −0.64 and −0.67, *P* < .01), and duration of the chewing sequence (*r* = −0.60 and −0.69, *P* < .01). There was no significant correlation between the percent yield and the maximum or mean bite force in either appetitive state. Palatability ratings were not significantly correlated with any masticatory variable in either the fasted or sated states.

The reliability of the masticatory outcome variables was uniformly high (all *P* < .001) as determined by intraclass correlation coefficients. The coefficients were mean particle size −0.68, mean percent yield −0.70, number of chews −0.98, maximum bite force −0.97, mean bite force −0.97, and chewing rate −0.90.

## 4. Discussion

Of particular interest in the present study were the effects of peanut flavor and processing properties on masticatory performance and proportional particle size distribution just prior to deglutition. The results of this study indicate that changes in the properties of peanuts through processing significantly influence masticatory parameters and lead to significant differences in particle size distribution at swallowing. However, these effects were not associated with palatability.

Based on previous research, suggesting palatability influences dimensions of chewing and eating behavior [17–20, 24], we hypothesized that changes in palatability in response to peanut processing would influence chewing efficiency and, as a result, particle size distribution. However, no significant associations were observed between palatability and either masticatory parameters or particle size distribution. The pattern of hedonic ratings and masticatory responses was comparable to that noted in an earlier study with almonds [16] suggesting, to some degree, that they are generalizable. While other studies have reported an increase in ingestion rate as a function of palatability [17–19, 22], rate of mastication was not directly measured. Ingestion rate in those studies was defined as the number of solid food units consumed per unit time, which is not a true measure of chewing rate. It is possible that palatability influences other dimensions of eating behavior not measured in the present study. 

Changes in variables other than palatability significantly altered parameters of mastication and particle size distribution of processed peanuts compared to unprocessed peanuts. Significantly less muscle work was needed to prepare the processed peanuts (honey roasted, roasted salted, and roasted unsalted) for swallowing compared to the unprocessed raw peanuts. The processed peanuts were also held in the mouth for a significantly shorter time compared to the raw peanuts. As a consequence, the processed forms all contained significantly larger particles in the preswallowed bolus compared to the unprocessed raw variety. A decrease in oral sensory stimulation as a function of a reduction in chewing duration could decrease the rate of development of sensory specific satiety and overall lead to an increase in food intake [25]. Further, swallowing larger particle sizes could lower the bioaccessibility of intracellular nut components such as unsaturated lipids, antioxidants, and phenolic compounds [7, 9], resulting in elevated losses of these cardioprotective components in the feces [8, 11].

This study indicates that the food particle size threshold level for swallowing varied significantly between processed and unprocessed forms and supports other reports that all food particles do not have to be reduced below a certain size or be reduced to a uniform particle size distribution before swallowing [14]. It also supports evidence that swallowed particle size is partly dependent on food form [13, 14, 16] and that additional oral cues must contribute to the initiation of deglutition. 

Food properties such as toughness/hardness, elasticity, and the percent water and fat affect chewing efficiency and final particle size [15, 26–28]. Processing of the peanuts leads to shrinkage and makes them more brittle and easier to fragment [16] than the raw unprocessed variety, possibly through modulating the percent water in the nut matrix. The water content of commercially available raw peanuts is approximately 6.5% while the values for dry roasted and honey roasted peanuts are approximately 1.5%–2%. These alterations may account for some of the variability noted in mastication between the processed and unprocessed peanut forms. Other alterations in nut properties, such as slicing, significantly influence mastication and particle size [16]. Consequently, it is evident that alterations of nut form during processing have significant implications for chewing parameters and swallowing particle size.

Earlier reports that appetitive state may influence ingestion rate [18] prompted us to investigate the impact of hunger and fullness sensations on mastication. It was previously reported that the ingestion rate (not necessarily chewing rate) increased transiently during the initial phase of a postdeprivation meal [18]. The present study observed effects of appetitive state on some masticatory parameters. Honey roasted, roasted salted, and raw peanuts were chewed at a significantly faster rate during the sated state compared to the fasted state. However, this did not alter the bolus particle size. A similar study with almonds also showed significant differences in masticatory parameters in the fasted compared to the sated state, and in agreement with our findings, this did not produce significant differences in particle size [16]. Based on these findings, the postingestive outcomes related to nut consumption are not expected to be altered by mastication during different appetitive states.

## 5. Conclusions

In conclusion, for the range of foods and properties tested here, palatability was not significantly associated with an alteration of the microstructural indices of chewing or the preswallowed bolus particle size distribution. However, many other varieties of peanuts are commercially available (e.g., Cajun, BBQ, wasabi flavored) and the impact of their sensory properties on masticatory function remains to be determined. Other properties imparted through processing were associated with the indices of mastication and resulting preswallowed particle size distribution. Whether these differences alter the health effects mediated by peanuts, possibly by altering the bioaccessibility of intracellular components, warrants further study. Of specific relevance in terms of cardiovascular health is whether the approved Food and Drug Administration health claim stating consumption of nuts (1 ounce (42 g) per day), as part of a diet low in saturated fat and cholesterol, may reduce the risk of heart disease [29], needs to be more specific in terms of nut form/processing.
